# Cancer-associated fibroblast subtype signature gene predicts survival and immunotherapy response in sarcoma

**DOI:** 10.1371/journal.pone.0353369

**Published:** 2026-07-20

**Authors:** Ji-Yong Sung, Jin-Hong Kim, Yi-Jun Kim

**Affiliations:** 1 Department of Biological Sciences, College of Natural Sciences, Seoul National University, Seoul, South Korea; 2 Department of Occupational and Environmental Medicine, College of Medicine, Ewha Womans University, Seoul, South Korea; 3 Graduate Program in System Health Science and Engineering, Ewha Womans University, Ewha Medical Research Institute, College of Medicine, Seoul, Republic of Korea; Sichuan University, CHINA

## Abstract

**Background:**

Sarcomas show heterogeneous responses to immune-checkpoint blockade (ICB), and cancer-associated fibroblasts (CAFs) are considered to shape the tumor immune microenvironment, yet CAF programs that predict ICB outcomes in sarcoma remain unclear.

**Methods:**

We investigated the interaction between different cell types in the sarcoma tumor microenvironment and their effects on immune-checkpoint blockade response, with a focus on identifying signature genes and molecular mechanisms that distinguish tumor-promoting from tumor-suppressive CAFs at the single-cell level. We analyzed single-cell data from two different sarcoma cohorts, transcriptome profiles of 206 sarcoma patients recruited from The Cancer Genome Atlas, and predicted immune-checkpoint blockade (ICB) response data inferred using the TIDE algorithm from 64 TCGA sarcoma patients.

**Results:**

We found 134 stem-like CAF-related signature genes in the recurrent group and eight signature genes in the metastasis group, defining three CAF subtypes (myofibroblastic CAF, antigen-presenting CAF, and inflammatory CAF). SIG134 and SIG8 were associated with TIDE-inferred ICB response in subtype-specific analyses: SIG134 in STLMS and ULMS, and SIG8 in MFS, STLMS, and ULMS. In addition, the MDK-NCL ligand-receptor signal transduction pathway was linked to the infCAF subtype and myoCAFs in metastatic sarcoma. Furthermore, SIG4 (*MDK*, *SDC2*, *LRP1*, and *NCL*) was highly expressed in inflammatory CAFs.

**Conclusions:**

Single-cell-derived CAF signatures may reflect sarcoma subtype-specific stromal programs associated with predicted ICB response and clinical outcome. SIG4 is proposed as a candidate prognostic signature that warrants further validation.

## Introduction

Precision medicine has enabled a paradigm shift in the treatment of patients with cancer, allowing for personalized treatment through the prediction of the exact drug and time [1]. Precision immunology integrates cutting-edge scientific understanding with evidence-based clinical practice to provide a holistic perspective on each individual patient’s condition [1]. Predicting the response of patients with cancer to immunotherapy remains a major challenge; however, there is currently no standard method to do so. As various cell types, cancer hallmark activities, and metabolic reprogramming occur [2] the tumor microenvironment [3] provides important clues for predicting immunotherapy response [4]. In addition, distinct metabolic profiles have been identified in tumors with high and low Epithelial-to-mesenchymal transition (EMT) activity [5]. Cancer-associated fibroblasts (CAFs) play a central role in the tumor microenvironment by depositing and remodeling the extracellular matrix, transmitting paracrine signals to neoplastic cells, and engaging in bidirectional cross-talk with infiltrating immune cells [6]. CAFs help cancer cells to grow and spread, create new blood vessels, trigger inflammation, and change the shape of the extracellular matrix (ECM) [7].

However, CAFs have also been hypothesized to inhibit the growth of pancreatic ductal adenocarcinoma (PDAC) [8]. Although the role of CAF is controversial, it is still known that CAFs activate tumor promotion. In a previous study on gastric cancer, we confirmed that prognosis-related gene signatures were enriched in CAF and found that *ACTA2* contributes to the mechanism of stem-like molecular subtypes [9]. Nevertheless, the function of CAFs in sarcomas remains largely unexplored.

With the advent of single-cell technology, the transcriptomic characteristics of CAFs across multiple cancer types have been characterized [10]. The results from these single-cell transcriptomic studies encourage the assumption that CAFs can be classified into three subtypes based on their myofibroblastic (myoCAF), inflammatory, and/or immune-regulatory (infCAF) and antigen-presenting (apCAF) activities [10]. Inflammatory CAFs are marked by IL6, Ly6c, and PDGFRA; immune-regulatory CAFs by Cxcl12; antigen-presenting CAFs by MHC class II (H2-Aa, H2-Ab1, and Cd74 in mice; HLA-DRA, HLA-DPA1, HLA-DQA1, and CD74 in humans); and myofibroblastic CAFs by ACTA2, TAGLN, and POSTN [10]. In this study, we analyzed the entropy transition of CAFs, their intercommunication, and favored energy metabolism at the single-cell level in sarcomas. We examined the tumor microenvironment at the single-cell level in an immunotherapy drug-resistant cohort. We also examined the signature genes of the CAF subtypes and the process by which CAFs were generated.

We identified a signature gene that predicts the immune response to immunotherapy and survival in a specific sarcoma subtype. Transcriptomic analyses can reveal CAF heterogeneity and identify biomarkers that can be used to develop new treatments.

## Methods

Abbreviations for sarcoma molecular subtypes used throughout this study are summarized in Table 1, following established WHO and TCGA sarcoma classifications.

**Table 1:** Glossary of sarcoma subtype abbreviations.

Abbreviation	Full name
UPS	Undifferentiated pleomorphic sarcoma
DDLPS	Dedifferentiated liposarcoma
MFS	Myxofibrosarcoma
SS	Synovial sarcoma
MPNST	Malignant peripheral nerve sheath tumor
STLMS	Soft tissue leiomyosarcoma
ULMS	Uterine leiomyosarcoma
OS	Osteosarcoma
CHS	Chondrosarcoma

### Single-cell RNA-seq analysis

We used single-cell cohorts of osteosarcoma [11] and synovial sarcomas [12]. CAF subtypes were categorized using the osteosarcoma cohort, with validation conducted across several cell types using the synovial sarcoma cohort. Single-cell RNA-seq data from osteosarcoma were obtained from the GEO database under accession GSE152048, which includes primary, recurrent, and metastatic tumor samples generated using the 10x Genomics platform. Single-cell RNA-seq data from synovial sarcoma were obtained from GSE131309 and were used for validation analyses.

Raw count matrices were processed using the Seurat R package. Cells with fewer than 200 detected genes or with mitochondrial gene expression exceeding 10% were excluded. Gene expression data were log-normalized and scaled prior to dimensionality reduction and clustering. When multiple samples were analyzed jointly, batch effects were corrected using Seurat’s integration workflow.

Fibroblasts and cancer-associated fibroblasts (CAFs) were identified based on the expression of canonical fibroblast markers, including COL1A1, COL1A2, DCN, and LUM, and by the absence of immune cell markers such as PTPRC (CD45). CAF subtypes were subsequently annotated based on established marker genes and cluster-level expression profiles. Non-fibroblast stromal cells and immune cells were excluded prior to downstream CAF-specific analyses.

CAF subtype labels were assigned based on established marker genes reported consistently in prior studies and were used to aid the biological interpretation of unsupervised clustering results, rather than to impose predefined or rigid cell state boundaries.

The default parameters of the “Seurat” R package were employed [13]. The R package “GSVA” [14] was used to calculate the scores of signature genes and pathway enrichment. High-entropy cells were identified using the StemID tool [15], and Gene Ontology analysis was conducted using signature genes and METASCAPE [16]. We utilized Cellchat [17] to identify the signaling pathway of ligand-receptor interactions for cell-cell communication among individual cells. The list of signature genes used for survival is provided in S1 Table. MDK (midkine), also commonly referred to as MK in the literature, is denoted using the official HGNC gene symbol MDK throughout this manuscript.

These genes were selected using the Seurat package to identify highly expressed genes in each CAF subtype based on a false discovery rate (FDR) of < 0.0001.

Signature scores were calculated as the mean z-score of the constituent genes within each predefined gene set and were applied consistently across all datasets. These scores were used to evaluate associations with survival outcomes and predicted immunotherapy response, rather than to construct optimized predictive models. Survival analyses were performed using predefined signature-based stratification without outcome-driven cutoff selection. Given the limited availability of clinical covariates and the sample size of the immunotherapy response cohort, formal multivariable regression modeling and performance metrics such as hazard ratios, C-index, or AUC were not assessed in this study. Therefore, the observed associations should be interpreted as indicative of prognostic relevance rather than definitive predictive performance, and further validation using multivariable models and independent cohorts will be required to establish clinical utility.

Signature derivation and validation were conducted using strictly separated datasets. SIG134 and SIG8 were derived exclusively from single-cell RNA-seq data based on entropy-defined CAF clusters using the StemID framework, without reference to survival outcomes, TCGA expression profiles, or immunotherapy response data. All parameters for entropy classification and gene selection were predefined and applied in an outcome-independent manner. Bulk RNA-seq datasets, including the TCGA sarcoma cohort and predicted immunotherapy response analysis, were used solely for downstream evaluation of the predefined signatures.

### Bulk RNA-seq analysis

We used two bulk RNA-seq cohorts: The Cancer Genome Atlas (TCGA) sarcoma and chondrosarcoma RNA-seq cohorts (E-MTAB-7264). FPKM normalization followed by log10 transformation was applied to bulk RNA-seq data to facilitate comparability across heterogeneous public datasets generated using different platforms and preprocessing pipelines. Importantly, the primary analyses in this study rely on relative within-cohort signature scores rather than absolute expression levels.

Survival analysis using TCGA sarcoma RNA-seq data was performed using gepia2 [18]. The immune-checkpoint blockade (ICB) response in the TCGA sarcoma cohort was analyzed using TIDE [19].

Immunotherapy response was computationally inferred using the Tumor Immune Dysfunction and Exclusion (TIDE) algorithm. TIDE predicts the likelihood of response to immune-checkpoint blockade based on gene expression signatures associated with T-cell dysfunction and exclusion, rather than observed clinical response outcomes. TCGA sarcoma samples with available bulk RNA-seq data were analyzed using the TIDE framework, and patients with complete transcriptomic profiles were included in the predicted responder and non-responder groups. No assumptions were made regarding specific immunotherapy regimens, RECIST-based response criteria, or treatment-time biopsy status.

After read mapping, it was processed in fragments per kilobase of transcript per million (FPKM) normalization and was transformed by log10. We performed a comparative study based on the expression levels of signature genes using bulk RNA sequencing of chondrosarcomas. Gene enrichment analysis was performed utilizing the “GSVA” R package [14]. We employed data from a previously published study on the immune tumor microenvironment deconvolution analysis of TCGA sarcoma data [20]. All statistics were based on a FDR < 0.001.

Multiple testing correction was applied separately within each analytical context, including differential gene expression analysis, pathway and gene set enrichment analyses, and immune cell correlation analyses, with false discovery rate (FDR) thresholds specified for each analysis.

For survival analyses, patients were stratified into high- and low-expression groups based on the mean value of the corresponding gene signature score within each cohort. Patients with expression values above the cohort mean were assigned to the high-expression group, whereas those with values below the mean were assigned to the low-expression group. This cutoff was predefined and applied uniformly across all analyses to ensure consistency and to minimize threshold-dependent bias.

For analyses involving multiple comparisons, including differential gene expression, pathway enrichment, and immune cell correlation analyses, p-values were adjusted using false discovery rate (FDR) correction as specified for each analysis. Kaplan–Meier survival analyses were performed using predefined gene signatures, and statistical significance was assessed using log-rank tests.

We used GSE202361, a publicly available bulk RNA-seq dataset from a phase 2 neoadjuvant immune-checkpoint blockade trial in soft-tissue sarcoma, to externally validate the predefined SIG134, SIG8, and SIG4 signatures. This cohort included patients with resectable retroperitoneal dedifferentiated liposarcoma (n = 17) and extremity/truncal undifferentiated pleomorphic sarcoma treated with nivolumab or nivolumab plus ipilimumab, with concurrent radiation therapy in the UPS cohort (n = 10). Processed TPM data from GEO were log-transformed, and signature scores were calculated as the mean z-score of constituent genes. Signature scores were compared across progressive disease, stable disease, and partial response groups using the Kruskal–Wallis test.

All bulk RNA-seq cohorts analyzed in this study were processed using cohort-appropriate normalized expression matrices, followed by log-transformation and within-cohort z-score standardization prior to signature scoring. Signature scores were calculated as the mean z-score of the constituent genes within each cohort, thereby avoiding direct comparison of absolute expression values across independent datasets.

### Ethics statement

This study used only publicly available, de-identified datasets. No new human participants were recruited, and no identifiable personal information was accessed. Therefore, additional institutional review board approval and informed consent were not required for this secondary analysis.

## Results

### Cancer-associated fibroblast subtypes reveal distinct gene signatures at the single-cell level

**Fig 1:**
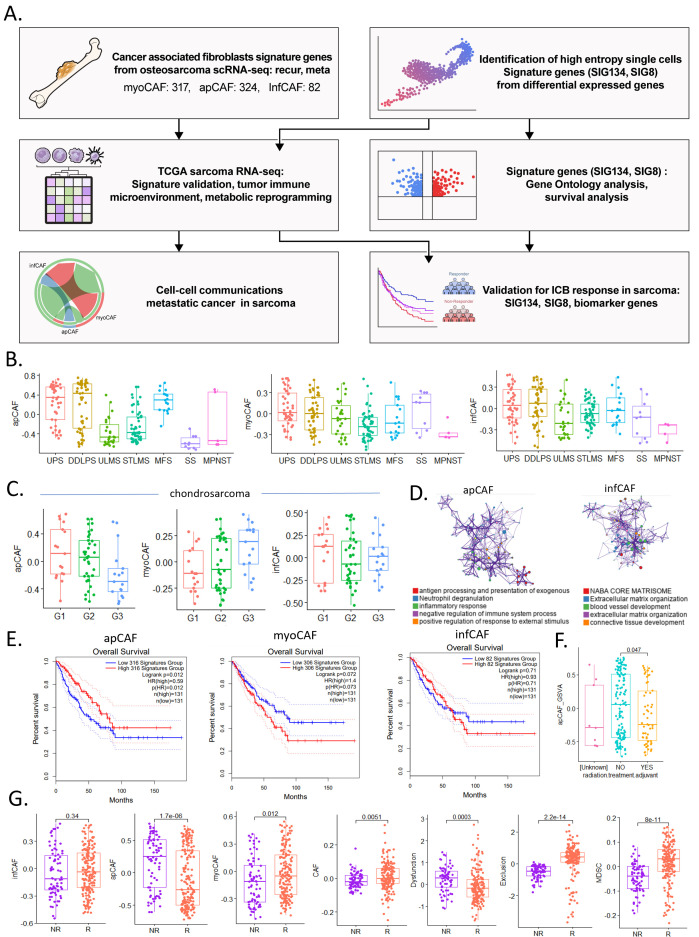
Cancer-associated fibroblast (CAF) subtypes reveal distinct gene signatures at the single-cell level. (A) Overview of the analysis pipeline. (B) Box plot for sarcoma’s CAF subtype signature in TCGA sarcoma molecular subtypes (UPS: undifferentiated pleomorphic sarcoma, DDLPS: dedifferentiated liposarcoma, ULMS: leiomyosarcoma – gynecologic, STLMS: leiomyosarcoma – soft tissue, MFS: myxofibrosarcoma, SS: synovial sarcoma, MPNST: malignant peripheral nerve sheath tumor). (C) Box plot for sarcoma’s CAF subtype signature in chondrosarcoma grade cohort. (D) Gene ontology network for apCAF signature and infCAF signature in metastatic osteosarcoma. (E) Kaplan–Meier plots showing the overall survival rates for the high and low CAF subtype signatures in TCGA sarcoma. (F) Boxplot for apCAF signature in radiation treatment adjuvant. (G) Boxplot for CAF signature and T-cell dysfunction, exclusion, MDSC between non-responder and responder in TCGA sarcoma.

Advancements in single-cell technology have enabled the differentiation of heterogeneous CAFs based on molecular characteristics. However, few studies have investigated the molecular characteristics of the CAF subtypes in sarcomas. We identified CAF subtype signature genes from osteosarcoma single-cell data and classified myoCAFs, infCAFs, and apCAFs using CAF marker genes [10] in the primary, recurrent, and metastatic osteosarcoma groups (Fig 1A). We identified 317 myoCAF signature genes, 82 infCAF signature genes, and 324 apCAF signature genes. We performed a gene enrichment test on the TCGA sarcoma RNA-seq data using CAF subtype genes found at the single-cell level. The apCAF signature was highly enriched in the undifferentiated pleomorphic sarcoma (UPS), dedifferentiated liposarcoma (DDLPS), and myxofibrosarcoma (MFS), whereas myoCAFs were enriched in the synovial sarcoma (SS). InfCAFs did not show any particularly enriched subtypes among the seven molecular subtypes (Fig 1B). We evaluated the CAF subtype signature using a chondrosarcoma cohort [21] and found that myoCAFs were significantly different as the grade increased, and apCAFs were highest in the low grades (Fig 1C). No discernible grading trend was observed for infCAFs.

In the metastatic cancer cohort, apCAFs were enriched in processes related to antigen processing, presentation of exogenous factors, neutrophil degradation, and inflammatory responses. Inf CAFs are enriched in the NABA core matrix, extracellular matrix architecture, and blood vessel formation (Fig 1D).

Additionally, we performed survival analysis using TCGA SARC data for each CAF subtype signature. The group with higher expression of the apCAF signature gene had a better prognosis. (*p* = 0.012). In contrast, groups with higher expression of myoCAF and infCAF signature genes had a poor prognosis, albeit not statistically significant (Fig 1E). Additionally, the group that did not undergo radiation treatment had a larger apCAF signature than the group that did (Fig 1F). The ability of these CAF signature genes to predict non-response (NR) and response (R) in the immunotherapy group was also assessed. Although the apCAFs of the NR group were noticeably greater, the results did not match the molecular subtype features, suggesting that the molecular subtype specificity of sarcomas is crucial. Furthermore, as anticipated, myoCAFs displayed the reverse outcome, with higher levels in the respondent group. Signatures related to T-cell dysfunction were significantly higher in the NR group (Fig 1G). It is feasible to deduce that the CAF signature gene is reflected in accordance with the molecular subtype characteristics because these traits do not reflect the molecular subtype.

### CAF subtypes within the sarcoma molecular subtype are associated with the tumor immune microenvironment

**Fig 2:**
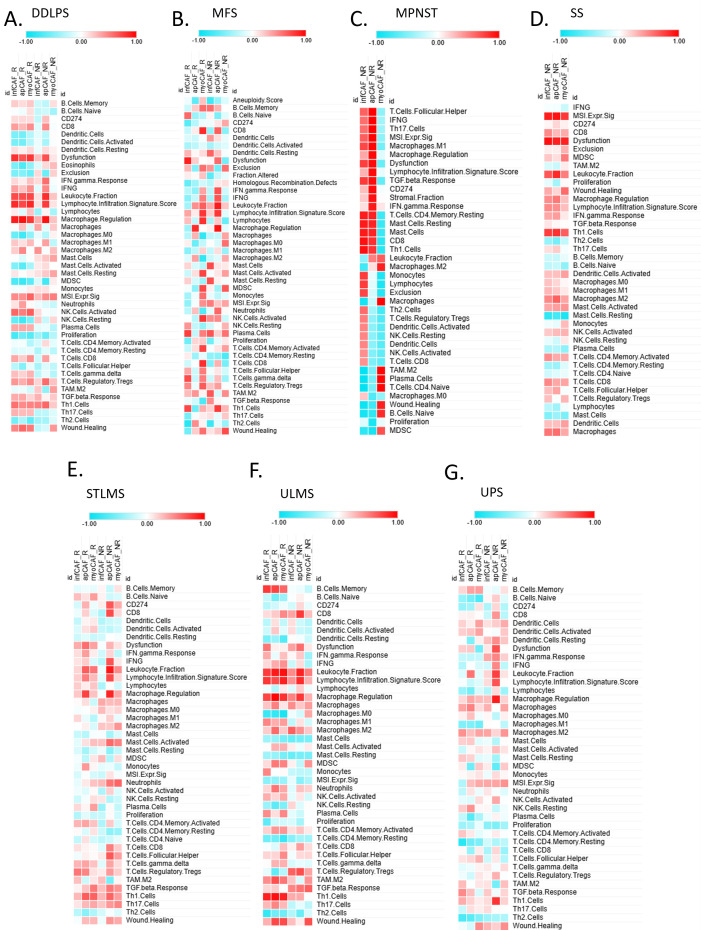
Correlation with immune cells and cancer-associated fibroblast (CAF) subtype signature in sarcoma molecular subtypes from immunotherapy outcome. (A) Heatmap of Pearson correlation coefficient in (A) dedifferentiated liposarcoma (DDLPS); (B) myxofibrosarcoma (MFS); (C) MPNST; (D) synovial sarcoma (SS); (E) leiomyosarcoma – soft tissue (STLMS); (F) leiomyosarcoma – gynecologic (ULMS); and (G) undifferentiated pleomorphic sarcoma (UPS).

Various tumor microenvironments are present in the sarcoma molecular subtypes. The capacity to induce T-cell activation is linked to MHC class II-expressing cells, known as antigen-presenting cells (APCs). Compared to other subtypes, SS was enriched in B-cell memory, natural-killer (NK) cell resting, mast cell activation, naïve T cells, and lymphocytes. However, T-cell CD8 was concentrated in the MPNST, MFS, and UPS, whereas naïve B cells were enriched in the MPNST. NK cells are activated in the SS and leiomyosarcoma – gynecologic (ULMS). We analyzed the correlation between the CAF subtype signature and immune cell types in the ICB non-responder and responder groups for each subtype. In general, a strong correlation with CD8 expression was observed in the responder group. In the R and NR groups, DDLPS displayed different relationships; in particular, the data from activated NK cells and plasma cells were contradictory (Fig 2A). T-cell gamma delta and Th17 cells were enriched in the responder group. The correlation in MFS displayed different features based on the CAF subtype characteristics rather than being differentiated by NR and R characteristics (Fig 2B). For example, only infCAFs in the responder group had a very high correlation with naïve B Cells, CD8 had a higher correlation with myoCAFs than with apCAFs in the responder group, and had a higher correlation with apCAFs in the non-responder group. In the MPNST, only the characteristics of the non-responder group were identified because of the small number of samples in the responder group (Fig 2C). CD8 positively correlated with infCAFs and apCAFs, and myoCAFs highly correlated with TAM M2, T Cells CD4 naïve, B cells, and macrophages. Although there were no responders in the SS group, the CAF subtypes in the NR group primarily displayed a comparable relationship (Fig 2D). For example, if all three CAF subtypes were highly correlated, the MSI expression signature, CD8 + T-cell dysfunction, leukocyte fraction, Th1 cells, macrophages M2, mast cells, activated NK cells, activated CD4 + memory activated T cells, CD8 dendritic cells, and macrophages. Instead of an instance in which NR and R exhibited contradictory associations, there was a propensity to rely on CAF subtype specificity in the leiomyosarcoma – soft tissue (STLMS) (Fig 2E). Compared to the R group, CD8 and CD274 showed a stronger positive correlation with apCAFs in the NR group. ULMS revealed that in both the R and NR groups, B-cell memory had an inverse relationship with CAF subtypes. Th17 cells were positively correlated with CAF subtypes in the responder group (Fig 2F). In the UPS, the correlation between apCAFs and CD8 and Th1 cells was highest in the non-responder group (Fig 2G). Although different immune cells exhibited linkages with CAFs, their characteristics varied based on the sarcoma subtype. Overall, apCAF expression positively correlated with T cells.

### Stem-like CAFs of different cancer types show CAF subtype heterogeneity based on the entropy state in sarcoma

**Fig 3:**
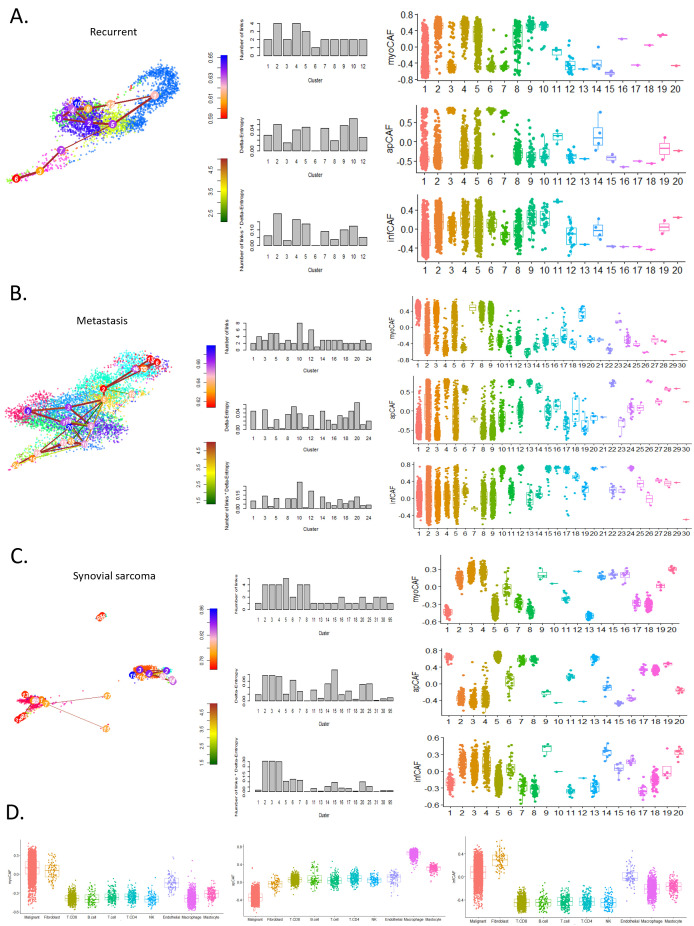
Cancer-associated fibroblast (CAF) subtype signature is enriched according to the entropy state. (A) tSNE plot for recurrent osteosarcoma (left); Bar graph of entropy in each cluster, top: number of links; middle: Delta-Entropy; bottom: number of links + Delta-Entropy) (middle), Boxplot for CAF subtype signature in entropy cluster (right). (B) tSNE plot for metastatic osteosarcoma (left); Bar graph of entropy in each cluster, top: number of links; middle: Delta-Entropy; bottom: number of links + Delta-Entropy) (middle), Boxplot for CAF subtype signature in entropy cluster (right). (C) tSNE plot for synovial sarcoma (left); Bar graph of entropy in each cluster, top: number of links; middle: Delta-Entropy; bottom: number of links + Delta-Entropy) (middle), Boxplot for CAF subtype signature in entropy cluster (right).

In the recurrent and metastatic groups of sarcomas, we identified the CAF subtype signatures that were enriched in high-entropy cell states. In the recurrent sarcoma group, clusters 10, 9, and 2 were identified as stem-like CAFs of the high-entropy group, and it was established that myoCAFs and infCAFs were primarily enriched. Clusters three and six of the low-entropy group were confirmed to have apCAF enrichment. The apCAF signature was mostly enriched in low-entropy cluster 6, whereas infCAF was enriched in high-entropy cluster 20 in the metastatic sarcoma group. Although apCAFs in a high-entropy state were not identified, this tendency demonstrates the traits of metastatic and recurring cancers, showing that this tumor microenvironment is conducive to tumor growth (Fig 3A and 3B). We also examined the distribution of signatures for each CAF subtype in single-cell data from synovial sarcoma. MyoCAFs are more enriched than infCAFs in the entropy clusters 15 and 3. Conversely, the low-entropy cluster was enriched in the apCAFs (Fig 3C). According to cancer type and tumor environment, there was a distinct difference in whether myoCAFs or infCAFs dominated the high-entropy cluster. These findings were consistent with those of an existing osteosarcoma single-cell cohort. In a synovial sarcoma cohort, we assessed this CAF subtype signature to determine whether it was expressed only in CAF or in other cell types. The myoCAF signature was most enriched in malignant cells; apCAFs were enriched in macrophages and mastocytes; and infCAFs were enriched in fibroblasts (Fig 3D). These findings suggest that the CAF transition reprograms the tumor microenvironment.

### Sarcoma’s novel CAF signature genes and metabolic reprogramming in sarcoma subtypes

**Fig 4:**
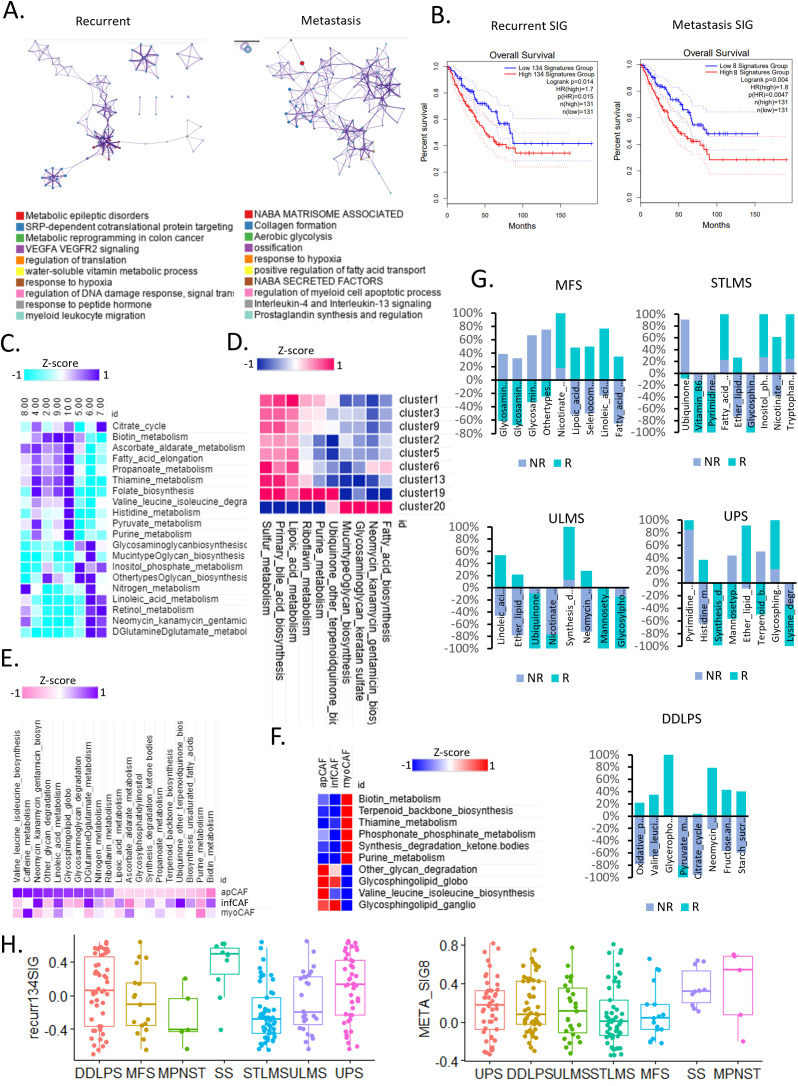
Identification of signature genes from high-entropy cells. (A) Gene ontology network for enriched genes in high-entropy Cancer-associated fibroblasts (CAF) in the recurrent group (left) and metastasis group (right). (B) Kaplan–Meier plots showing the overall survival rates for the differentially expressed gene in high-entropy CAFs. SIG134 (left), SIG8 (right). (C) Enriched metabolic reprogramming pathway in different molecular subtypes in TCGA sarcoma (myxofibrosarcoma (MFS), leiomyosarcoma – soft tissue (STLMS), leiomyosarcoma – gynecologic (ULMS), undifferentiated pleomorphic sarcoma (UPS), dedifferentiated liposarcoma (DDLPS)) (D) Heatmap of highly activated metabolic pathway in recurrent stem-like clusters (E) Heatmap of highly activated metabolic pathway in metastatic stem-like clusters (F) Heatmap of highly activated metabolic pathway in CAF subtypes from the recurrent group. (G) Bar plots showing metabolic pathway enrichment patterns across predicted ICB non-responder and responder groups according to sarcoma molecular subtype. (H) Boxplot for enriched signature genes in multiple molecular subtypes in TCGA sarcoma (SIG134) (left), (SIG8) (right).

Genes enriched in cells belonging to the high-entropy group were subjected to gene ontology analysis. Gene ontology enrichment terms reported here were automatically generated by the analysis tool based on curated databases, and disease-associated terminology reflects standardized annotations rather than author-defined labels. Metastatic and recurrent groups exhibited different traits. Matrisome-associated NABA and collagen-production pathways were enriched in the metastatic group, whereas metabolism-related pathways and the SRP-dependent cotranslational protein-targeting pathway were prominent in the recurrent group (Fig 4A). To further confirm survival in the TCGA SARC cohort, we selected eight genes in the metastatic group and 134 genes in the recurrent group based on an FDR < 0.0001 by differential expression analysis of genes that varied in the high-entropy group. Patients with high expression of SIG134 (*p* = 0.014) and SIG8 (*p* = 0.004) had a poor prognosis (Fig 4B).

To better understand the type of energy metabolism carried out by cells in the high-entropy group, we examined 83 KEGG metabolic pathways. We also verified that low-entropy cells in the recurrence group showed abundant glycosaminoglycan production. The low-entropy cells in the recurrent group were mainly apCAFs, and it was discovered that these apCAFs were important for glycan synthesis (Fig 4C). In the case of the metastatic group, another metabolic heterogeneity was confirmed, and high-entropy cells were enriched in the fatty acid synthesis pathway (Fig 4D). On the basis of this metabolic heterogeneity, we infer that apCAF-associated metabolic programs differ between recurrent and metastatic osteosarcoma. In recurrent osteosarcoma, apCAFs exhibited a relatively active energy-metabolism profile, whereas this metabolic activity was attenuated in metastatic osteosarcoma. These findings suggest that metastatic osteosarcoma may be associated with a distinct stromal metabolic state compared with recurrent disease (Fig 4E and 4F). We extended these results and confirmed the heterogeneity of metabolic reprogramming in each immunotherapy response group according to the sarcoma subtype (Fig 4G).

We validated the increased activity of metabolic pathways in the ICB NR and R groups based on the molecular subtype in the TCGA sarcoma dataset. Consequently, four metabolic pathways in the NR group were enriched in MFS. (glycosaminoglycan biosynthesis-chondroitin sulfate/dermatan sulfate, glycosaminoglycan biosynthesis-keratan sulfate, glycosaminoglycan biosynthesis-heparan sulfate/heparin, other types of O-glycan biosynthesis) (Fig 4G).

In contrast to the other MFS subtypes, we discovered that the non-responder group had increased glycan metabolism. The responder STLMS group was enriched in fatty acid degradation, whereas the ULMS group was enriched in ketone degradation. In the non-responder group, UPS accumulated in the pyrimidine metabolic pathway. DDLPS was mainly enriched in metabolic reprogramming in the responder group. Depending on the sarcoma subtype and immunotherapy response, this metabolic heterogeneity displays distinct heterogeneities. Using the sarcoma subtype, we examined the novel signatures 134 and SIG8. SIG8 and SIG134 were highly expressed in MPNST and SS, respectively (Fig 4H).

### Signature genes predict survival and immunotherapy response

**Fig 5:**
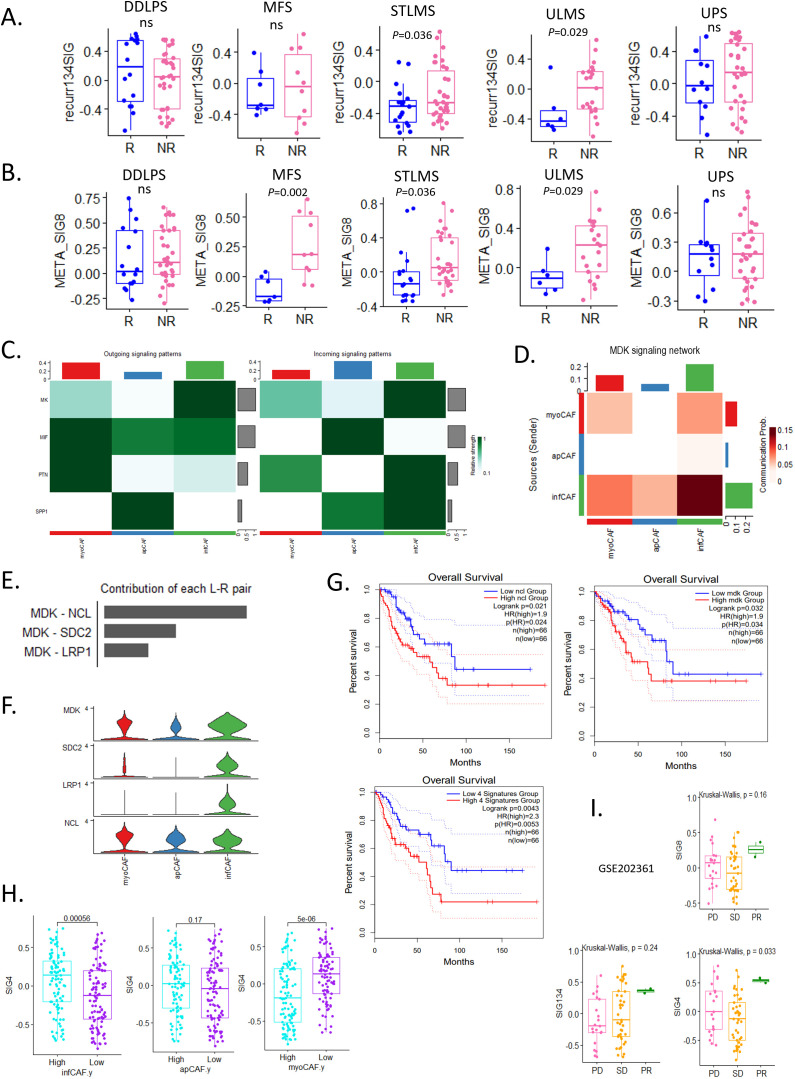
Validation of signature genes (SIG134, SIG8, and SIG4) in multiple molecular subtypes. (A) Boxplot of SIG134 in molecular subtypes (B) Boxplot of SIG8 in molecular subtypes (C) Ligand-receptor signaling in metastatic Cancer-associated fibroblast (CAF) subtypes. (D) MDK signaling network (E) Expected ligand-receptor pair (F) Four gene expression (MDK, SDC2, LRP1, NCL) in CAF subtype in the metastatic group. (G) Kaplan–Meier plots showing the overall survival rates for the high and low MDK, NCL, the four signature genes (MDK, SDC2, LRP1, NCL) expression in TCGA sarcoma. (H) Boxplot of SIG4 in high and low CAF subtype signature in TCGA sarcoma. (I) Boxplot of SIG134, SIG8, SIG4 for immune-checkpoint blockade response cohort: progressive disease (PD), stable disease (SD), partial response (PR).

We identified SIG134 and SIG8 in the NR and R groups according to the sarcoma subtype. SIG134 was significantly upregulated in the non-responder group for STLMS and ULMS, while SIG8 was significantly upregulated in the non-responder group for MFS, STLMS, and ULMS (Fig 5A and 5B). Given the clinical relevance of metastatic disease, we examined predicted cell-cell interactions in metastatic cancer using the previous metabolic analysis. Except for the apCAF-targeting approach, MDK-NCL signaling was significant, and the ligand-receptor interaction between myoCAFs and infCAFs was confirmed (Fig 5C–5E). Therefore, four genes related to ligand-receptor interaction were selected as signature genes. MDK (HR = 1.9, p(HR)=0.034; log-rank *p* = 0.032) and NCL (HR = 1.9, p(HR)=0.024; log-rank *p* = 0.021) were associated with a poor prognosis in the high-expression group, and the group with high expression of the four signature genes (HR = 2.3, p(HR)=0.0053; log-rank *p* = 0.0043; Fig 5G) had a poor prognosis (Fig 5G). Four genes were particularly highly expressed in infCAFs, and we confirmed that they were expressed at significantly higher levels in the high infCAF group regardless of the subtype in the TCGA SARC cohort (Fig 5H). We re-evaluated SIG134, SIG8, and SIG4 based on ICB response outcomes from actual clinical trials instead of the predicted values. In the external GSE202361 ICB-treated sarcoma cohort, predefined SIG134, SIG8, and SIG4 scores were compared across PD, SD, and PR groups. SIG4 showed a significant difference across response groups, whereas SIG134 and SIG8 showed weaker or non-significant trends (Fig 5I). These findings support the applicability of these signatures to bulk transcriptomic data.

To further evaluate the prognostic relevance of the identified CAF-related markers, we integrated SIG134, SIG8, and SIG4 and established a final set of 145 unique genes (SIG145) after removing duplicated genes. We constructed SIG145 as an exploratory composite score to determine whether these independently derived but biologically related CAF-associated signatures collectively reflect a broader stromal transcriptional program with prognostic relevance. In the TCGA sarcoma cohort, univariate Cox proportional hazards regression using the primary mean-based cutoff showed a directionally higher risk of death in patients with a high SIG145 score, although this association did not reach statistical significance (Hazard Ratio [HR] = 2.55, 95% Confidence Interval [CI] = 0.82–7.87). As a sensitivity analysis, we performed Kaplan–Meier survival analysis using a median-based cutoff instead of the primary mean-based cutoff applied in the main analyses. Patients in the high-SIG145 group showed poorer overall survival than those in the low-SIG145 group (log-rank p = 0.043; S1 Fig). Thus, while the mean-cutoff Cox model should be interpreted cautiously, the directionally consistent Cox estimate and the median-cutoff Kaplan–Meier analysis support the potential prognostic relevance of SIG145 in sarcoma. Nevertheless, SIG145 remains an exploratory aggregate rather than an independently derived prognostic signature, and these findings should be interpreted with caution.

## Discussion

Diverse CAF subsets are preserved in many organs, cancer subtypes, and animals. The composition of CAFs can vary among several cancer subtypes within the same organ. Comparable marker genes were detected across various cancer types, including IL6, Ly6c, and PDGFRA, indicating infCAFs; Cxcl12, representing immune-regulatory CAFs; MHC class II (H2-Aa, H2-Ab1, and Cd74 in murine models; HLA-DRA, HLA-DPA1, HLA-DQA1, and CD74 in humans), indicating apCAFs; and ACTA2, TAGLN, and POSTN, representing myoCAFs.

The interpretation of antigen-presenting CAFs (apCAFs) requires particular caution, as this population is primarily defined by the expression of MHC class II–related genes. In this study, apCAF-like stromal cells were annotated based on the co-expression of MHC class II genes together with canonical fibroblast markers and low expression of immune lineage markers. However, we acknowledge that the absence of explicit doublet detection and ambient RNA correction represents a limitation, and that immune-derived transcripts may contribute to MHC class II signals in some stromal populations.

Consistent with this limitation, we observed that apCAF-associated signatures were also enriched in macrophages and mast cells in the synovial sarcoma cohort, suggesting that these signatures may not be strictly CAF-specific when broadly applied. Accordingly, we adopt a more conservative terminology and refer to this population as “MHC class II–high stromal cells” in contexts where fibroblast lineage identity cannot be unambiguously resolved.

Furthermore, in bulk transcriptomic analyses, apCAF-associated signatures should be interpreted as composite signals that may partially capture immune antigen-presenting cell activity in addition to fibroblast-derived expression. These findings highlight the complexity of stromal–immune transcriptional overlap in sarcoma and underscore the need for cautious biological interpretation. Future studies incorporating orthogonal validation approaches and refined single-cell preprocessing strategies will be required to more precisely delineate the lineage identity and functional role of MHC class II–expressing stromal populations.

The tumor microenvironment greatly affects the proliferation and progression of cancer cells. The efficacy of immunotherapy depends on the interaction between immune cells and the tumor microenvironment. The response to immunotherapy in sarcoma is poorer than that in other malignancies, necessitating the identification of novel biomarkers to predict immunotherapy efficacy.

The current study examined the relationship between immune cells and CAFs in the tumor microenvironment and proposes single-cell-derived candidate transcriptomic signatures (SIG134, SIG8, and SIG4) associated with subtype-dependent predicted immunotherapy response in sarcoma. Based on the cancer-specific aspects of the CAF subtype, we identified a sarcoma-specific CAF subtype. For example, in gastric cancer, immune-regulatory CAFs are highly enriched in stem cell-like types and contribute to poor patient outcomes. An insufficient response to anti-PD-L1 therapy in PDAC has been associated with TGF-β-driven LRRC15 + CAFs [22]. Sarcoma can be classified into a variety of molecular subtypes, each of which has a distinct tumor microenvironment and different interactions with immune cells.

MyoCAFs contribute to the initial phases of cancer development. They are primarily involved in collagen deposition, ECM remodeling, smooth muscle contraction, wound healing, cancer cell growth, and metastasis. InfCAFs are involved in inflammation, immune cell recruitment and suppression, cancer cell activation, and growth [10]. ApCAFs are involved in T-cell activation, T-cell recruitment, and infiltration. This CAF heterogeneity reorganizes the ECM in the tumor microenvironment according to their mission, inflammation, and regulation of the immune system, together with direct effects on cancer cell growth and metastatic dissemination.

We found that infCAFs were associated with MDK–NCL signaling, and that SIG134 and SIG8 showed significant associations with differential immunotherapy response in STLMS and ULMS. These characteristics have been validated by the presence of immune cells inside the tumor immune microenvironment and the heterogeneity of cancer-associated fibroblasts, which generate distinct tumor microenvironments based on their relationships with each other. The heterogeneity of CAFs significantly influences immunotherapy responses and offers insights into the appropriate treatment of patients from a precision medicine standpoint.

We acknowledge that the statistical power of some analyses is limited, particularly those involving immunotherapy response data and subtype-specific subgroup comparisons with small sample sizes. Therefore, results derived from these analyses should be interpreted with caution, and statistically marginal associations should be considered exploratory rather than conclusive.

We acknowledge that Pearson correlation may not fully account for the compositional nature of immune cell estimates, and alternative metrics such as rank-based correlations could be explored in future studies.

The MDK–NCL signaling axis highlighted in this study should be interpreted within the context of hypothesis generation based on computational ligand–receptor inference. CellChat analysis provides predictions of putative intercellular communication and does not demonstrate physical protein–protein interactions, downstream signaling activation, or functional consequences. Accordingly, the MDK-NCL association identified here is not presented as a validated mechanistic pathway, but rather as a plausible signaling hypothesis emerging from transcriptomic patterns associated with CAF heterogeneity in sarcoma.

Consistent with the bioinformatics-based nature of this work, experimental validation of MDK–NCL signaling and its potential role in immune modulation or immunotherapy resistance lies beyond the scope of the present study. Instead, these findings are intended to serve as an informational resource to guide future mechanistic and experimental investigations.

Similarly, SIG4 is proposed as a transcriptomic signature associated with prognosis, rather than as a mechanistically validated biomarker. Taken together, these results emphasize the value of ligand–receptor inference as a framework for generating biologically relevant hypotheses from complex tumor microenvironment data, while underscoring the importance of cautious interpretation in the absence of direct functional validation.

## Supporting information

S1 Fig Kaplan–Meier survival curves for the combined 145-gene signature (SIG145) in TCGA sarcoma. Patients were stratified into high- and low-expression groups based on the cohort mean.
(DOCX)

S1 Table Subtype marker genes. (XLSX)
