# Supplementary material for: Cancer-associated fibroblast subtype signature gene predicts survival and immunotherapy response in sarcoma
Source: PLoS One. 2026 Jul 20;21(7):e0353369. doi: 10.1371/journal.pone.0353369 (PMC13384308; doi:10.1371/journal.pone.0353369)
Supplement: S1 Fig — Patients were stratified into high- and low-expression groups based on the cohort mean. (DOCX) [file pone.0353369.s001.docx]

**Supplementary material**


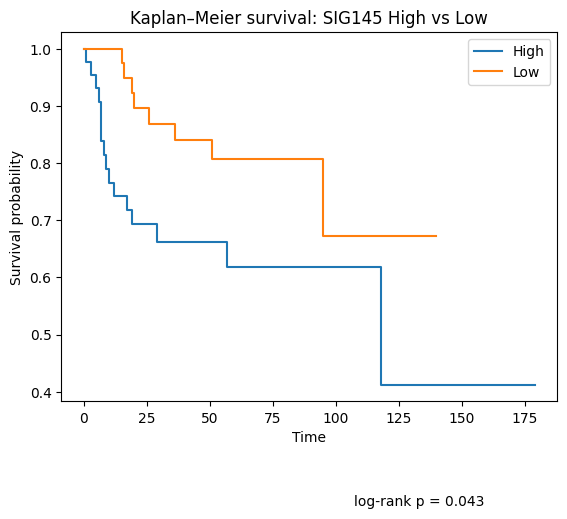


**Supplementary Figure S1.** Kaplan–Meier survival curves for the combined 145-gene signature (SIG145) in TCGA sarcoma. Patients were stratified into high- and low-expression groups based on the cohort mean.
